# Addison's Disease Mimicking Cerebral Salt Wasting Syndrome: A Case of Severe Hyponatremia

**DOI:** 10.7759/cureus.117262

**Published:** 2026-09-30

**Authors:** Somarajan Anandan, Sajeesh Rajendran, Joesni Joy, Anandhu Suresh, Caroline S Pushpaletha

**Affiliations:** 1 Neurology, St. Joseph's Mission Hospital, Anchal, IND; 2 Neurology, Mar Sleeva Medicity, Palai, IND; 3 Medicine, St. Joseph's Mission Hospital, Anchal, IND

**Keywords:** addison's disease, cerebral salt wasting syndrome, hyponatremia, primary adrenal insufficiency, pseudohypothyroidism, renal salt wasting

## Abstract

Hyponatremia is a common electrolyte abnormality with varying degrees of severity. It can be asymptomatic if mild and can cause convulsions and coma if severe. Systematic investigation of hyponatremia helps to find out the etiology and proper management. Here, we are presenting the case of a 67-year-old man who presented with vomiting, hyperpigmentation, and hypovolemic hyponatremia due to Addison's disease. Evaluation of hyponatremia showed elevated urinary sodium as expected in Addison's disease, but there was hypouricemia with increased fractional excretion of uric acid suggestive of cerebral salt wasting syndrome. Even with the correct diagnosis, wrong medication interval can cause inadequate rise in serum sodium. Mild thyroid-stimulating hormone elevation can occur in untreated isolated Addison's disease. Addison's disease should always be considered in unexplained hyponatremia even if laboratory findings are suggestive of cerebral salt wasting syndrome. Hyponatremia in Addison's disease may respond poorly to hypertonic saline until glucocorticoid deficiency is corrected, and steroid and fluid replacement is essential and lifesaving.

## Introduction

Hyponatremia is the most common electrolyte disturbance in hospitalized patients with a reported incidence of up to 35% [1]. It can be mild, moderate, or severe. Depending on the patient's volume status, hyponatremia can be hypovolemic, euvolemic, or hypervolemic. Even though there is hyponatremia, total body sodium can be low (in hypovolemic), normal (in euvolemic), and high (in hypervolemic). Hyponatremia is essentially a disorder of water balance with excess water in relation to total body sodium [2]. Correct classification of hyponatremia is essential to find out the etiology and management. Some conditions causing hyponatremia have overlapping biochemical features but diametrically opposite therapeutic strategies, and wrong diagnosis can result in potential therapeutic consequences. In certain diseases like Addison's disease, steroid replacement alone can correct hyponatremia. Primary adrenal insufficiency (Addison's disease), first described by Thomas Addison in 1855, usually affects the whole adrenal cortex and leads to deficiencies of cortisol, aldosterone, and adrenal androgen to a variable extent. Most cases (75-80%) are attributed to autoimmune adrenalitis and are often associated with other autoimmune disorders. Less frequent causes of primary adrenal insufficiency include infections (tuberculosis and acquired immunodeficiency syndrome (AIDS)), metastatic carcinoma, and several medications [3].

Cortisol inhibits antidiuretic hormone (ADH) secretion, and cortisol deficiency in Addison's disease enhances ADH secretion. In Addison's disease, aldosterone deficiency also contributes to hyponatremia by causing sodium wasting and hypovolemia. Characteristic electrolyte abnormality in Addison's disease is hyponatremia with hyperkalemia, but hyperkalemia is often absent (in 30-50% of cases) and mimics other causes of hypovolemic hyponatremia with the exception that urinary sodium and chloride are inappropriately high. Other conditions causing hyponatremia with elevated urinary sodium are syndrome of inappropriate antidiuresis (SIAD), cerebral salt wasting syndrome (CSWS), salt-losing nephropathy, and diuretics [4].

Addison's disease usually causes hypovolemic hyponatremia with elevated urinary sodium levels. It is usually associated with pre-renal azotemia, hyperuricemia, and low fractional excretion of uric acid. Our patient had hypovolemic hyponatremia with hypouricemia and high fractional excretion of uric acid mimicking CSWS.

## Case presentation

A 67-year-old man presented to the emergency department with a one-day history of multiple episodes of vomiting and fatigue. There was no history of headache, vertigo, abdominal pain, diarrhea, polyuria, oliguria, or fever. He had a few episodes of giddiness and one episode of syncope after admission. He was a diabetic on metformin. He has a history of darkening of the skin for the last 4-5 years. There was no other significant past medical history. He was not on any other medications. Examination showed generalized hyperpigmentation of the skin, palmar creases, cheeks, and tongue (Figure 1). His blood pressure was 110/70 mmHg, and his pulse rate was 70/minute. His tongue was moist, and skin turgor was normal. There was a mild postural drop in systolic blood pressure of 15 mmHg, and his heart rate increased by 26 beats. His abdomen was soft and non-tender. The rest of the examination was unremarkable. His hematological evaluation showed eosinophilia and normocytic normochromic anemia (Table 1). He had hypoosmolar hyponatremia with high urine osmolality, hypouricemia, high urinary sodium, and elevated fractional excretion of uric acid suggestive of SIAD or CSWS (Tables 2-3). His postural syncope and mild postural drop in blood pressure favored a hypovolemic hyponatremia. Urine was sent before giving 3% saline as part of the hyponatremia panel. In view of history of hyperpigmentation, random serum cortisol was ordered. His random serum cortisol level was 1.56 microgram/dL, and next-day morning (8 AM) serum cortisol was 1.15 microgram/dL even after a dose of hydrocortisone (100 mg) the previous evening. Multiple boluses of 3% saline (400 mL) and water restriction did not improve his serum sodium levels; instead, his sodium levels fell to 101 mEq/L by the second day. His intravenous hydrocortisone dose was changed from 100 mg twice daily to 50 mg every six hours. 3% saline was given thrice daily along with one liter of normal saline, with close monitoring of serum sodium levels and urinary output, with a target rise in sodium of <8 mEq/L on the first day and 6 mEq/L on subsequent days. With this regimen, his sodium levels gradually improved to 127 mEq/L by the eighth day (Table 4). His symptoms subsided in four days. His serum adrenocorticotropic hormone (ACTH) was markedly elevated, and his serum dehydroepiandrosterone sulphate (DHEA-S) was markedly reduced, confirming the diagnosis of primary adrenal insufficiency. His thyroid-stimulating hormone (TSH) was mildly elevated with normal free T4 levels. On the sixth day of admission, his eosinophilia subsided (eosinophils: 0%), and TSH normalized without any levothyroxine supplement. His chest X-ray did not show any evidence of tuberculosis. He was discharged with hydrocortisone tablets (10 mg in the morning and 5 mg in the evening) and fludrocortisone 100 mcg per day. At review, 15 days after the patient's discharge, his serum sodium was 139 mEq/L, his potassium was 4.1 mEq/L, and he was asymptomatic.

**Figure 1:**
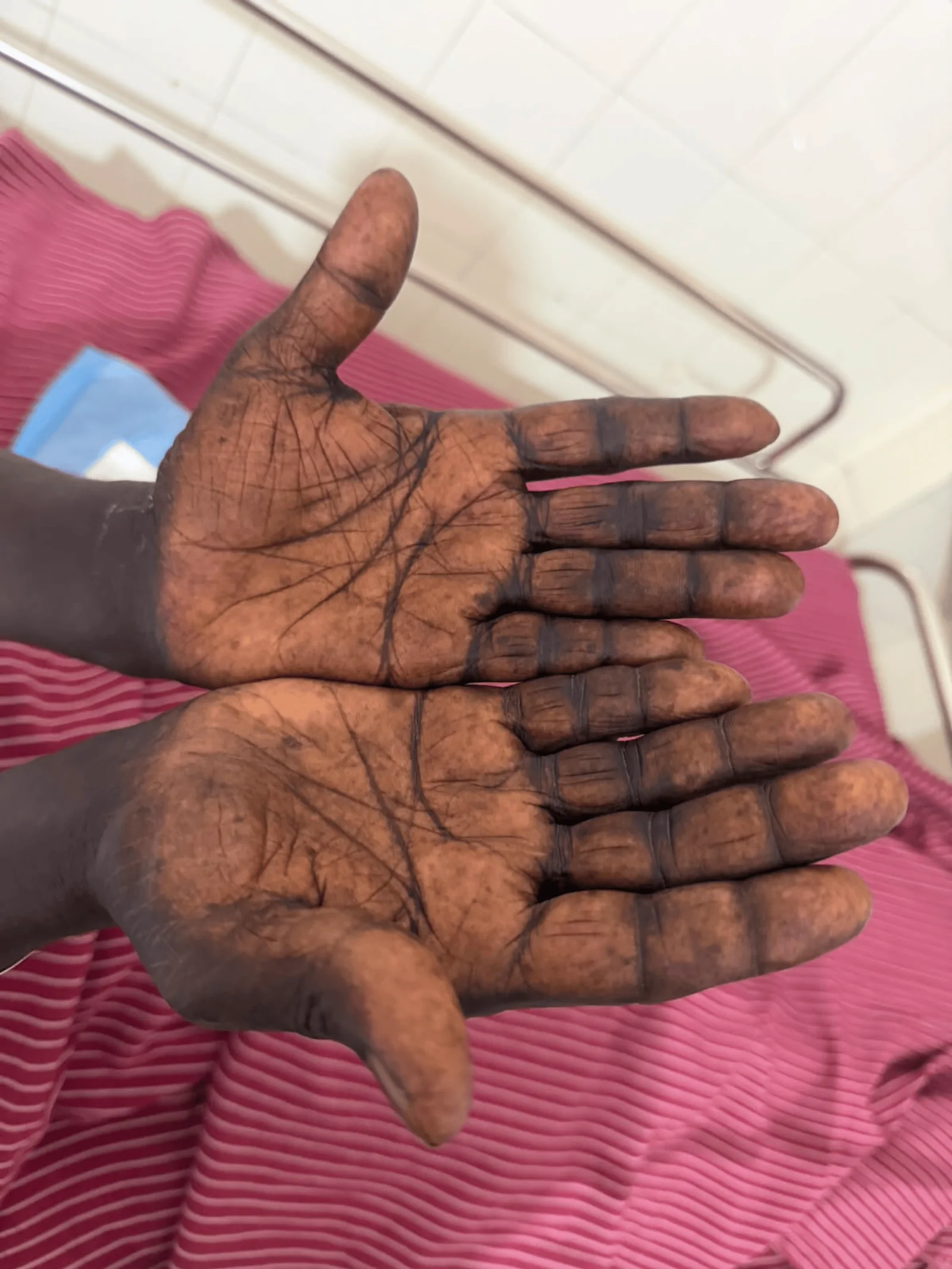
Hand showing hyperpigmentation of palmar and digital creases

**Table 1:** Hematological parameters

Blood parameters	Day 1	Day 6	Normal range
Hemoglobin	12.5 gm/dL	10.1 gm/dL	13-16.5 gm/dL
Total count	4720 cells/mm^3^	6300 cells/mm^3^	4000-11000 cells/mm^3^
Neutrophils	58%	92%	40-70%
Lymphocytes	33%	8%	20-40%
Eosinophils	9%	0%	1-6%
Platelet count	1.99 lakhs/mm^3^	1.64 lakhs/mm^3^	1.5-4.5 lakhs/mm^3^
Erythrocyte sedimentation rate	16 mm/hour	25 mm/hour	<34 mm/hour

**Table 2:** Blood biochemistry and hormonal evaluation

Parameters	Result	Normal value
Fasting blood glucose	85 mg%	70-110 mg%
Urea	19 mg%	20-45 mg%
Creatinine	0.6 mg%	0.7-1.3 mg%
Sodium	106 mEq/L	136-145 mEq/L
Potassium	4.2 mEq/L	3.5-5.5 mEq/L
Magnesium	1.4 mg/dL	1.8-2.2 mg/dL
Chloride	76 mEq/L	96-106 mEq/L
Bicarbonate	19 mmol/L	22-26 mmol/L
Calcium	8.8 mg%	8.5-10.5 mg%
Phosphorus	3 mg%	2.5-4.5 mg%
Uric acid	1.6 mg%	3.4-7 mg%
Serum osmolality	230 mOsm/Kg	280-295 mOsm/Kg
Thyroid-stimulating hormone	6.565 mIU/mL	0.4-4.5 mIU/mL
Free T4	0.87 ng/dL	0.8-1.8 ng/dL
Cortisol (8 AM)	1.15 microgram/dL	5-25 microgram/dL
Adrenocorticotropic hormone	752 pg/mL	7.2-63.3 pg/mL
Anti-thyroid peroxidase antibody	0.45 mIU/mL	<5.61 mIU/mL
Dehydroepiandrosterone sulphate	11.2 microgram/dL	33.6-249 microgram/dL

**Table 3:** Urine electrolytes and osmolality

Parameters	Result	Normal value
Urine sodium	191 mEq/L	>20 mEq/L
Urine potassium	29 mEq/L	25-125 mEq/L
Urine chloride	185 mEq/L	110-250 mEq/L
Urine creatinine	44 mg/dL	20-320 mg/dL
Urine uric acid	17.3 mg/dL	250-750 mg/dL
Urine osmolality	538 mOsm/Kg	50-1200 mOsm/Kg
Fractional excretion of sodium	2.46%	1-2%
Fractional excretion of potassium	9.4%	10-20%
Fractional excretion of uric acid	14.74%	4-11%

**Table 4:** Serum sodium during the hospital stay and recommended sodium correction per day

Blood parameter	D1	D2	D3	D4	D8
Serum sodium	106 mEq/L	101 mEq/L	108 mEq/L	114 mEq/L	127 mEq/L
Recommended sodium correction	Up to 8 mEq/L	Up to 6 mEq/L	Up to 6 mEq/L	Up to 6 mEq/L	-

## Discussion

About 2.7-3.8% of unselected patients presenting to the emergency room with euvolemic hyponatremia have undiagnosed adrenal insufficiency, and it is as high as 15% among patients admitted to specialized units for the evaluation of hyponatremia [5]. In a series, 40% of patients aged 65 years or older presenting with hyponatremia had pituitary-adrenal dysfunction [6]. Classical electrolyte abnormality in Addison's disease is hyponatremia with hyperkalemia (Table 5). The diagnosis of primary adrenal insufficiency may be difficult to establish since hyperkalemia may be absent in approximately 30-50% of patients. Low sodium (<137 mEq/L) is seen in 84%, elevated TSH in 52%, and high potassium (>5 mEq/L) in 34% of cases. Elevated TSH with hyponatremia should trigger the consideration of Addison's disease [7]. SIAD is the most common cause of euvolemic hyponatremia. Even though SIAD has well-established clinical and laboratory criteria, it is a diagnosis of exclusion as hypothyroidism and hypoadrenalism should be ruled out. Even though CSWS is a cause of hypovolemic hyponatremia, clinical differentiation between mild hypovolemia and euvolemia is a challenge.

**Table 5:** Metabolic abnormalities in Addison's disease

Metabolic abnormalities
Hyponatremia
Hyperkalemia
Hypercalcemia
Hyperuricemia
Metabolic acidosis
Azotemia
Hypermagnesemia [8]
Hypoglycemia
Elevated thyroid-stimulating hormone

As cortisol suppresses TSH, cortisol deficiency itself can elevate TSH, leading to the erroneous diagnosis of hypothyroidism (pseudohypothyroidism). As autoimmune thyroid disease is the most common association of Addison's disease, clinicians should be careful in diagnosing hypothyroidism in the setting of Addison's disease. In Addison's disease with co-existing hypothyroidism, always give hydrocortisone before thyroxine to avoid worsening of hypocortisolism or even precipitating an adrenal crisis [9].

The most common cause of primary adrenal insufficiency/Addison's disease relates to the autoimmune destruction of the adrenal cortex. Antibodies against the 21-hydroxylase enzyme are seen in approximately 90% of patients. As other autoimmune diseases are common with Addison's disease, one should search for other autoimmune diseases. Autoimmune thyroid disease is the most common endocrine comorbidity in autoimmune Addison's disease. Other common associated autoimmune diseases are type 1 diabetes mellitus (15%), B12 deficiency (15%), vitiligo (17%), primary gonadal deficiency (8%), and celiac disease (5%) [10]. Addison's disease and at least one additional autoimmune condition, such as autoimmune thyroid disease or type 1 diabetes mellitus, are features of autoimmune polyendocrine syndrome type 2 (APS-II) [11].

Addison's disease usually causes hypovolemic hyponatremia with elevated urinary sodium and can mimic CSWS (Table 6). It is characterized by hyperuricemia and low fractional excretion of uric acid. In contrast, secondary adrenal insufficiency is almost indistinguishable from SIAD and should be ruled out in all SIAD without obvious etiology. Secondary adrenal insufficiency is characterized by low ACTH levels, alabaster-like pale skin, and absence of hyperkalemia (Table 7).

**Table 6:** Addison's disease vs. cerebral salt wasting syndrome FEUA: fractional excretion of uric acid FEUA % = (Urine uric acid × S. creatinine) × 100/(S. uric acid × Urine creatinine) Table Credit: [12]

Features	Addison's disease	Cerebral salt wasting syndrome
Volume status	Hypovolemic	Hypovolemic
Sodium level	Hyponatremia	Hyponatremia
Potassium level	Hyperkalemia	Normal or low
Uric acid	High	Low
FEUA	Normal or low	High
Blood glucose	Can be low	Normal

**Table 7:** Addison's disease vs. adrenocorticotropic hormone deficiency Table Credit: [12]

Features	Addison's disease	Adrenocorticotropic hormone deficiency
Volume status	Hypovolemic	Euvolemic
Sodium level	Hyponatremia	Hyponatremia
Potassium	Hyperkalemia	Normokalemia
Adrenocorticotropic hormone level	High	Low
pH	Metabolic acidosis	Respiratory alkalosis [13]
Hypoglycemia	Possible	Common
Skin pigmentation	Hyperpigmentation	Alabaster-like pale skin
Fractional excretion of uric acid	Normal or low	High

CSWS is defined as a renal loss of sodium during intracranial disorders leading to hyponatremia and a decrease in extracellular fluid volume. It is believed that natriuretic factors such as atrial natriuretic peptide (ANP), brain natriuretic peptide (BNP), C-type natriuretic peptide (CNP), and dendroaspis natriuretic peptide (DNP) may play a role in CSWS [14]. It closely mimics SIAD biochemically [15]. CSWS is more appropriate as there are well-documented cases without cerebral disease. The major difference between CSWS and SIAD is that the former is a cause of hypovolemic hyponatremia and the latter is a cause of euvolemic hyponatremia (Table 8). Even though CSWS is a hypovolemic hyponatremia, pre-renal azotemia is absent [16]. Even though Addison's disease and CSWS are characterized by hypovolemic hyponatremia with increased urinary sodium loss, Addison's disease is associated with low fractional excretion of uric acid [17,18]. 

**Table 8:** Cerebral salt wasting syndrome vs. syndrome of inappropriate antidiuresis Table Credit: [13]

Parameters	Cerebral salt wasting syndrome	Syndrome of inappropriate antidiuresis
Extracellular fluid volume	Low	Normal
Urine sodium	Elevated	Elevated
Renin	Elevated	Normal
Aldosterone	Elevated	Normal
Serum uric acid	Low	Normal to low
Fractional excretion of uric acid	High	High
Fractional excretion of phosphate	High	Normal
Fractional excretion of uric acid after sodium correction	High	Normal

Elevated fractional excretion of uric acid is seen in SIAD, CSWS, and some cases of thiazide-induced hyponatremia. Our patient had features suggestive of CSWS with high urinary sodium, low serum uric acid, and high fractional excretion of uric acid. Addison's disease is usually associated with normal uric acid or hyperuricemia, and fractional excretion of uric acid is low. In addition, there is usually elevated blood urea due to hypovolemia. 

Cortisol inhibits ADH secretion, and cortisol deficiency in Addison's disease enhances ADH secretion. In Addison's disease, aldosterone deficiency also contributes to hyponatremia by causing sodium wasting and hypovolemia. Aldosterone deficiency is responsible for hyperkalemia which is present in many cases. DHEA-S is the most abundant steroid hormone in the circulation, and almost all is secreted by the zona reticularis of the adrenal cortex. It is often reduced in Addison's disease. Because of the lack of diurnal variation, DHEA-S blood sampling can be done at any time [19]. If morning serum cortisol is 3-15 microgram/dL, an ACTH stimulation study should be done to rule out adrenal insufficiency [20].

Hyponatremia with severe symptoms (vomiting, seizure, coma) should be treated with 3% saline [21]. Diagnosing adrenal insufficiency is very important in this setting as hyponatremia may respond poorly to 3% saline. This underpins the importance of looking for subtle clinical evidence of Addison's disease [22].

Hypomagnesemia is not a feature of Addison's disease. Our patient had hypomagnesemia and probable mild metabolic acidosis. We could not find out the etiology of hypomagnesemia. His recurrent vomiting or long-term undisclosed proton pump inhibitor use is speculated to be the cause of hypomagnesemia. Our patient had normocytic normochromic anemia and eosinophilia which is a common association in Addison's disease (Table 9). His fall in hemoglobin and neutrophilia are probably secondary to hemodilution and steroid effect, respectively.

**Table 9:** Hematological abnormalities in Addison's disease Table Credit: [23]

Blood picture
Normocytic normochromic anemia
Eosinophilia
Relative lymphocytosis
Neutropenia

A systematic biochemical approach using a hyponatremia panel is very useful in classifying and finding out the etiology of hyponatremia, and a hyponatremia panel is suggested (Table 10).

**Table 10:** Suggested hyponatremia panel Table Credit: [24]

Blood	Urine
Random blood sugar	Urine osmolality
Urea	Sodium
Serum osmolality	Potassium
Sodium	Uric acid
Potassium	Phosphate
Uric acid	Creatinine
Phosphate	Chloride
Creatinine	
Bicarbonate	
Thyroid-stimulating hormone	
Free T4	
Cortisol (8 AM)	

Our study has a few limitations. We could not prove the cause of primary adrenal insufficiency as autoimmune adrenalitis as we could not do a 21-hydroxylase antibody test and computed tomography of the abdomen due to poor financial status. Even though there are no features of tuberculosis elsewhere, we could not exclude adrenal tuberculosis. We did not obtain plasma aldosterone levels and urinary magnesium studies to document hypoaldosteronism and to find out the etiology of hypomagnesemia, respectively.

## Conclusions

Hyponatremia is a common electrolyte abnormality. Here, we describe a case of severe hyponatremia due to Addison's disease which biochemically mimicked CSWS. Even though there is salt wasting in Addison's disease, hypouricemia and high fractional excretion of uric acid suggested CSWS. As cortisol inhibits TSH secretion, Addison's disease can be associated with mild elevation of TSH without any primary thyroid disease.

Addison's disease should always be considered in unexplained hyponatremia even if the laboratory features point to CSWS. Serum cortisol should be done in all cases of unexplained hypovolemic or euvolemic hyponatremia. Cortisol and fluid replacement corrects hyponatremia. Even after the correct diagnosis and right dose of steroids, wrong dosage interval may result in inadequate rise in serum sodium. Treat cortisol deficiency before thyroid hormone replacement to avoid precipitating an Addisonian crisis.
